# Unplanned hospital presentations in oncology patients receiving chemotherapy: a secondary analysis of a randomized controlled trial to explore opportunities for improving supportive care

**DOI:** 10.1007/s00520-026-10510-7

**Published:** 2026-03-13

**Authors:** Bora Kim, Chantale Boustany, Judith Fethney, Judy M. Simpson, Kate White

**Affiliations:** 1 Susan Wakil School of Nursing and Midwifery, University of Sydney, Camperdown, NSW 2006 Australia; 2 The Daffodil Centre, The University of Sydney, and Cancer Council NSW, Camperdown, NSW 2006 Australia; 3 Cancer Care Research Unit, Sydney Local Health District, Camperdown, NSW 2050 Australia; 4 Mental Health Research Unit, South Western Sydney Local Health District, Liverpool, NSW 2170 Australia; 5 School of Medicine, Western Sydney University, Campbelltown, NSW 2560 Australia; 6 Sydney School of Public Health, Camperdown, NSW 2006 Australia

**Keywords:** Chemotherapy, Unplanned presentation, Emergency department, Hospital admission, Oncology, Neoplasm

## Abstract

**Purpose:**

This study aimed to report the incidence, common reasons, and associated risk factors for unplanned hospital presentations during chemotherapy treatment.

**Methods:**

A secondary analysis using data from a randomized controlled trial containing hospital data for the first three cycles of chemotherapy of adult oncology patients in two tertiary hospitals in Australia. Descriptive statistics were used to report hospital utilization patterns. Poisson regression explored risk factors for unplanned presentations.

**Results:**

Analyses included data from 346 patients; 115 patients (33%) made one/or more presentations during the first three cycles of chemotherapy. Of 144 unplanned presentations, 74 (51%) were made during cycle 1. Predominant reasons were fever with/without neutropenia (*n* = 50, 35%) and nausea/vomiting (*n* = 30, 21%). Fifty-two percent (*n* = 75) of unplanned presentations did not result in hospital admission. Of 346 patients, 70 (20%) had hospital admissions with a median length of stay of 3 days (IQR 2–7). Multivariable analysis identified the following as predictors for unplanned presentations: cancer stage (stage 1 vs stage 4: IRR 2.50, 95% CI, 1.28–4.89; P = 0.01) and cancer type (lung cancer vs breast cancer: IRR 2.25, CI, 1.26–4.01; P = 0.01).

**Conclusion:**

Nausea/vomiting management support may be one area warranting improvement, a frequent reason for unplanned presentations that are potentially preventable. Such support will be most beneficial during the first treatment cycle, when most unplanned presentations occurred. A high proportion of unplanned presentations did not result in hospital admission, indicating an opportunity to manage some of the side effects within primary care or outpatient settings, rather than utilizing emergency department services.

## Introduction

Chemotherapy is a crucial aspect of cancer treatment. An estimated 9.8 million people globally required chemotherapy during the course of their illness in 2018 [1]. In Australia, approximately 68,942 patients received chemotherapy in 2020 [2]. Despite its wide use, chemotherapy is known for a range of common side effects, including but not limited to nausea, vomiting, diarrhea, oral mucositis, and neutropenia [3], with more than 75% of Australian patients experiencing multiple moderate or severe side effects during their chemotherapy treatment [3]. These side effects can also lead to secondary complications such as febrile neutropenia, weakness, electrolyte imbalances and hypovolemia [4], conditions frequently associated with unplanned hospital presentations [5].

A recent systematic review based on studies from multiple countries reported that approximately 44% of cancer patients present to the emergency department within one year of diagnosis [5]. This aligns with findings from Australian studies, which similarly show that around 40% of patients receiving systemic treatment experience unplanned hospital presentations [6, 7]. In Australia, cancer-related hospital admissions accounted for 1 in 9 hospital visits, with approximately 1.3 million hospitalizations occurring in 2019–2020 [8]. Of these, approximately 74% of cases were discharged on the same day, with chemotherapy side effects being a major factor. Although many of these presentations are inevitable, some of the common reasons for presentations, such as nausea, vomiting, diarrhea, and constipation [9], are potentially preventable through the effective use of evidence-based pharmacological and supportive measures, such as anti-emetic, anti-diarrheal medications, and nutritional support [10–12].

According to global projections, it is estimated that the number of patients requiring chemotherapy will significantly increase by 53% between 2018 and 2040 [1]. The rise in cancer populations and expected growth in care demands [13] underscore the necessity for targeted support to high-risk groups that are more prone to poor side effects management and deterioration. Previously reported risk factors include being underweight [14], high-dose chemotherapy [14], overseas-born status [14], certain cancer types [14], younger age [15], non-white race [15] and lower education level [15]. However, the heterogeneity of predictors included across studies makes it challenging to reach a consensus.

Over the past two decades, a number of studies have examined the incidence and risk factors associated with unplanned hospital presentations during systemic cancer treatment [16]. However, a recent systematic review has highlighted methodological limitations in the existing literature [16]. For example, studies that included data based on a specific time periods (e.g. 1 year) rather than a treatment specific follow-up period risk missing hospital events among participants who enter the dataset late in the observation window. Follow-up durations also varied widely, from one month to three years, potentially distorting incidence estimates, as longer observation periods increase the chance of hospital visits. Additionally, when a fixed follow-up period is used, variations in treatment cycle length and associated effects may not be adequately accounted for; for example, risk may be overestimated in patients receiving shorter-cycle treatments, as they undergo more cycles within the same timeframe compared to those on longer regimens. Failing to adjust for at-risk days, such as in patients with prolonged inpatient stays during which unplanned presentations cannot occur, may lead to underestimation of their risk. Addressing some of these limitations, the current study aimed to (1) report the incidence and common reasons for unplanned hospital presentations and admissions during the first three cycles of chemotherapy, and (2) identify clinical and sociodemographic risk factors associated with unplanned presentations.

## Materials and methods

### Study design and setting

This study used longitudinal patient data obtained from a prospective, randomized controlled trial (RCT) conducted between August 2015 and January 2019 in two tertiary teaching hospitals with specialist cancer centers in Sydney, Australia [9]. The primary objective of the RCT was to investigate the impact of home-based chemotherapy support, delivered by community nurses using a shared care model, on the number of unplanned presentations during the first three chemotherapy cycles. The group assignment was not considered in the current analyses, as the intervention and control groups showed similar clinical outcomes, demonstrated by no statistically significant difference between the two groups in the number of unplanned presentations made. As part of routine care, all patients received pre-chemotherapy education and were advised to contact the treatment unit if they felt unwell. Patients receiving treatment with palliative intent were referred to a community-based multidisciplinary palliative care team for additional support. The study has been reported in accordance with the Strengthening the Reporting of Observational Studies in Epidemiology (STROBE) guide [17].

### Sample

The analysis included 346 oncology trial participants aged 18 years and above, who had commenced their first cycle of outpatient chemotherapy for solid tumors and were residents of a suburb within the participating health districts. Exclusion criteria of the trial were inability to provide informed consent in English, concurrently receiving radiotherapy, or receiving targeted therapies only without chemotherapy. These criteria were established to ensure the findings remain relevant to the management of regimes containing chemotherapy. Additionally, patients undergoing radiotherapy attend the cancer center daily for treatment, which may influence the likelihood of making unplanned presentations. Patients commencing their first cycle of chemotherapy were identified and screened by clinical staff or the trial coordinator. Written informed consent was collected from all study participants.

### Ethics

The study obtained ethical approval from the appropriate hospital research integrity committee (Royal Prince Alfred Hospital Zone protocol X13-0101). All procedures were performed in compliance with the approved study protocol.

### Data collection

#### Outcome variables

The outcome variable included the number of unplanned hospital presentations during the first three cycles of chemotherapy (from day 1 of the first cycle to the last day of cycle 3). Unplanned visits to the cancer center or emergency department were documented using an electronic medical record (eMR) review. Data were collected on the incidence of unplanned hospital presentations and admissions, reasons for these clinical episodes, and hospital length of stay. Each clinical episode could have more than one reason documented. This information was then verified with patient-reported hospital utilization data collected by research staff at the end of each cycle. When hospital visits occurred outside the two study sites, patient-reported hospital utilisation data were used in the analysis (*n* = 6).

As the study aimed to examine the incidence and risk factors for unplanned hospital presentations during the first three cycles of chemotherapy, all patients who initiated chemotherapy and consented were included in the analysis, regardless of whether they completed all three cycles. For those who discontinued treatment early, hospital presentation data were collected up to the final cycle received. This approach reflects real-world treatment patterns and allows for the inclusion of patients who discontinued treatment early, capturing outcomes during the period they were at risk (i.e., while receiving treatment) to provide a pragmatic estimate of early treatment-related hospital presentations. Additionally, for patients who commenced concurrent radiotherapy, data were collected up to the last chemotherapy cycle received without radiotherapy.

#### Predictor variables

Demographic and clinical data were used as predictors. At baseline, patients’ self-reported sociodemographic and clinical data were collected, including marital status, education level, primary language, country of origin, working status and whether they had previously received chemotherapy treatment. Clinical data, including age, biological sex, cancer type and stage, and treatment intent, were collected via eMR review. The types of chemotherapy regimes were also recorded from the eMR at the first cycle, and emetogenic risk (high, moderate, low) [18] were used as one of the clinical predictors. The full list of categorizations of each variable is presented in Table 1.

**Table 1:** Characteristics of the participants at baseline

	Total (*N* = 346)		Unplanned presentation		Unplanned hospitalisation	
Characteristics	n 346	(%) 100.0	n 115	(%) 33.2	n 70	(%) 20.2
Gender						
Male	123 (35.6)		50 (43)		35 (50)	
Female	223 (64.5)		65 (57)		35 (50)	
Age Mean (SD) (range)	59.3 (13.1) (24–87)		59.9 (13.9) (24–81)		62.4 (13.7) (33–81)	
Marital status						
Never married	54 (15.6)		16 (14)		8 (11)	
Married/de facto	236 (68.2)		84 (73)		48 (69)	
Separated/divorced	30 (8.7)		8 (7)		6 (9)	
Widowed	20 (5.8)		4 (3)		5 (7)	
Missing	6 (1.7)		3 (3)		3 (4)	
Primary language						
English	261 (75.4)		79 (69)		45 (64)	
Other than English	74 (21.4)		29 (25)		19 (27)	
Missing	11 (3.2)		7 (6)		6 (9)	
Primary language						
Born outside Australia	147 (42.5)		51 (44)		34 (49)	
Born in Australia	187 (54.0)		57 (50)		30 (43)	
Missing	12 (3.5)		7 (6)		6 (9)	
Education						
Primary school only/year 10 or below	55 (15.9)		24 (21)		17 (24)	
Year 12/HSC^a^ or equivalent	38 (11.0)		13 (11)		9 (13)	
TAFE^b^ certificate or diploma (or trade qualification)	63 (18.2)		16 (14)		6 (9)	
University undergraduate degree	77 (22.3)		27 (23)		13 (19)	
Higher degree/postgraduate	64 (18.5)		17 (15)		9 (13)	
Missing	49 (14.2)		18 (16)		16 (23)	
Working status						
Currently working	130 (37.6)		31 (27)		15 (21)	
Not in paid work	47 (13.6)		15 (13)		11 (16)	
Retired	128 (37.0)		56 (49)		35 (50)	
Missing	41 (11.8)		13 (11)		9 (13)	
Type of cancer						
Breast	97 (28.0)		29 (25)		14 (20)	
Colorectal	50 (14.5)		9 (8)		5 (7)	
Genito-urinary	33 (9.5)		11 (10)		6 (9)	
Gynaecological	57 (16.5)		15 (13)		10 (14)	
Lung	41 (11.8)		22 (19)		17 (24)	
Upper gastrointestinal	50 (14.5)		22 (19)		14 (20)	
Other	18^c^ (5.2)		7 (6)		4 (6)	
Cancer stage						
I	13 (3.8)		8 (7)		4 (6)	
II	55 (15.9)		14 (12)		6 (9)	
III	77 (22.3)		23 (20)		9 (13)	
IV	169 (48.8)		59 (51)		44 (63)	
Missing	32 (9.2)		11 (10)		7 (10)	
Intent of treatment						
Curative	145 (41.9)		46 (40)		20 (29)	
Palliative	124 (35.8)		48 (42)		36 (51)	
Not specified	77 (22.3)		21 (18)		14 (20)	
History of previous chemotherapy						
Received chemotherapy before	57 (16.5)		14 (12)		7 (10)	
First chemotherapy treatment	283 (81.8)		99 (86)		62 (86)	
Missing	6 (1.7)		2 (2)		1 (1)	
Emetic risk						
Low	60 (17.3)		15 (13)		9 (13)	
Moderate	198 (57.2)		67 (58)		44 (63)	
High	88 (25.4)		33 (29)		17 (24)	

^a^ Higher School Certificate

^b^ Technical and Further Education

^c^ 5 × Mesothelioma, 3 × Peritoneal, 4 × Head and Neck, 2 × Metastatic adenocarcinoma, 2 × Sarcoma, 1 × Brain cancer, 1 × Unknown primary

### Missing data

There were no missing data for hospital utilization and chemotherapy regime. For demographic and clinical data, missing values were replaced using multiple imputations with predictive mean matching and 30 imputations.

### Data analysis

IBM SPSS Statistics V28 was used to analyse the data. The sample size calculation was performed based on the primary outcome, as described in detail elsewhere [9]. The incidence of unplanned presentations and hospital admissions were reported using descriptive statistics. These included the frequency and percentages of unplanned presentations at each cycle, hospital length of stay for those admitted, and reasons for presentations and hospital admissions. A sensitivity analysis of the incidence of unplanned presentations was conducted using complete cases of patients who received all three cycles of chemotherapy. A two-sample z-test was used to compare the proportion of patients who made unplanned presentations in the full sample versus the complete-case sample.

To identify risk factors for unplanned presentations, we used the total number of unplanned presentations during cycles 1, 2, and 3 per patient as the outcome variable. We then examined baseline clinical and demographic predictors using Poisson regression. Univariable regressions involving each predictor were first conducted, and predictors with any category having P < 0.20 were included in multivariable regression, followed by progressive backward removal of statistically non-significant variables to arrive at a final model containing variables with P < 0.05. Both univariable and multivariable tests were adjusted for the hospital site (due to differences in local management processes), and the natural logarithm of time at risk was used as the offset variable to account for varying treatment lengths, as well as periods when making unplanned presentations was not possible (e.g., hospitalization, death).

To calculate the time at risk of making an unplanned presentation, we aggregated the time from the start of cycle 1 to (1) the last day of cycle 3, (2) the last day meeting the eligibility criteria, or (3) the last day of being alive. If a patient had a hospital admission during a cycle, the length of stay was subtracted from the time at risk.

## Results

A total of 346 patients enrolled in this trial, out of 2,159 eligible patients screened; mean age 59 (range 24–97), female (*n* = 223, 64%), stage 4 cancers (*n* = 169, 49%), curative intent (*n* = 145, 42%). The baseline demographic and clinical characteristics of the complete cohort are summarized in Table 1. Ten patients (2.9%, mean age 69.1, range 54–76) died during the study period. Of those, 8 and 2 had stage 4 and 3 cancers, respectively. The number of participants at each cycle and the reasons for withdrawal are reported elsewhere [9].

Most patients (*n* = 228, 66%) received 21 days cycle treatment regime, followed by 14 days cycle (*n* = 78, 23%), 28 days cycle (*n* = 31, 9%) and 7 days (*n* = 9, 3%). The majority (*n* = 316, 91%) completed all three cycles of chemotherapy, while 11 (3%) completed only the first cycle and 19 (6%) completed up to the second cycle. Forty-seven patients (14%) experienced treatment delays greater than 7 days, 24 (51%) of which were for unexpected clinical reasons such as treatment toxicity, disease progression, and hospitalization. The median time at risk for unplanned hospital presentations was 63 days (IQR: 44–65, range: 3–112).

 The mean age of the 115 individuals who made unplanned presentations was 60 years (SD, 13.9; range, 24–81). Table 1 presents the baseline characteristics of those who made unplanned presentations. Among all 346 patients, 231 (67%) made no unplanned presentations, while 115 (33%) patients made 144 unplanned presentations. Out of 115 who made presentations, 91 patients (79%) made one unplanned presentation, 20 (17%), 3 (3%) and 1 (1%) patients made two, three and four presentations, respectively. Among all 346 patients, 65 (19%), 39 (11%), and 28 (8%) made unplanned presentation(s) during cycles 1, 2, and 3, respectively. The sensitivity analysis showed that 30% of patients (96/316) in the complete-case sample made unplanned presentations, compared to 33% in the full sample (95% CI: −4.0, 10.1).

**Fig. 1:**
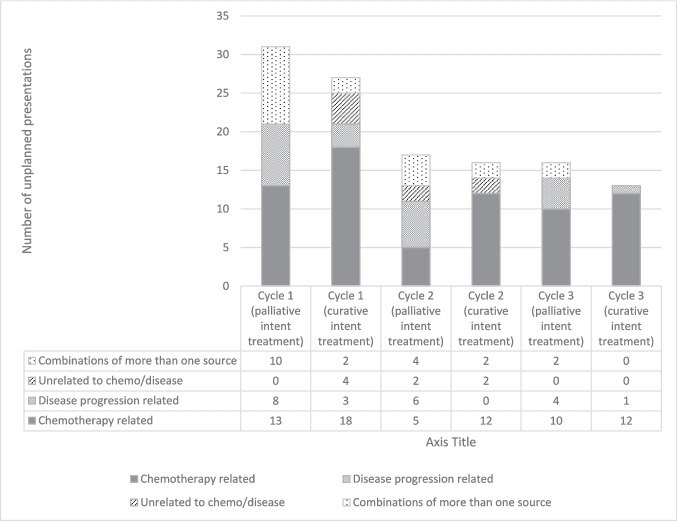
Proportion of the main causes for unplanned presentations by chemotherapy treatment cycle and treatment intent*. *Baseline treatment intent was missing in 21 patients in the study cohort, so the figure contains 120 unplanned presentations made by 94 patient records who had baseline treatment intent available

Among the 144 unplanned presentations, more were observed during cycle 1 (*n* = 74, 51%) than in subsequent cycles (cycle 2: *n* = 40, 28%; cycle 3: *n* = 30, 21%). 81 (56%) were chemotherapy-related, followed by 29 disease-related (20%), 23 combinations of chemotherapy- and disease-related (16%) and 11 not related to chemotherapy or disease (8%). Figure 1 illustrates the proportion of main causes of unplanned presentations, calculated from the total number of presentations at each chemotherapy cycle, and grouped according to patients’ baseline treatment intent. Those receiving curative intent treatment had proportionally more chemotherapy treatment-related unplanned presentations (palliative vs curative: 42% vs 67% in cycle 1, 29% vs 75% in cycle 2, 63% vs 92% in cycle 3).

The most common reason for unplanned presentations was fever either with or without neutropenia (*n* = 50, 35%), followed by nausea and vomiting (*n* = 30, 21%) and abdominal pain (*n* = 21, 16%) (Table 2). Seventy-five (52%) unplanned presentations were discharged without leading to hospital admission.

**Table 2:** Reasons for unplanned presentations

	Unplanned presentation *N* = 144^a^/346
	*n* (%)
Fever without neutropenia	27 (18.8)
Fever with neutropenia	23 (16.0)
Nausea and vomiting	30 (20.8)
Abdominal pain	21 (15.6)
Cough/sore throat	17 (11.8)
Diarrhoea	14 (9.7)
Shortness of breath	13 (9.0)
Lethargy/Fatigue	12 (8.3)
Constipation	11 (7.6)
Chest pain	9 (6.3)
Dehydration	7 (4.9)
Anxiety	7 (4.9)
Headache	7 (4.9)
Confusion	6 (4.2)
Collapse	5 (3.5)

^a^ Numbers add up to more than 144 as some patients presented with multiple symptoms

Of 346 patients, 70 (20%) had 86 hospital admissions, 11 of which were direct admissions without prior unplanned presentation. The median length of stay irrespective of reason was 3 days (IQR 2–7, range 1–39 days), and the most common reasons for hospital admissions were: fever (*n* = 44, 51%), nausea and vomiting (*n* = 17, 20%), respiratory infection (*n* = 15, 17%) and abdominal pain (*n* = 12, 14%). The median length of stay for the ten most common reasons for hospitalization was less than 7 days (Table 3).

**Table 3:** Reasons for unplanned hospital admissions and length of stay

Reason for admission^a^	*N* = 86^a^/346 *N* (%)	Length of stay^b^ Median (IQR)
Fever with neutropenia	26 (30.2)	3.5 (2.75–4.75)
Fever without neutropenia	18 (20.9)	3.0 (2.0–9.25)
Nausea and vomiting	17 (19.8)	3.0 (1.75–7.75)
Respiratory infection	15 (17.4)	6.0 (2.0–14.0)
Abdominal pain	12 (14.0)	3.0 (2.0–7.0)
Diarrhoea	12 (14.0)	4.0 (3.0–9.25)
Constipation	11 (12.8)	3.0 (2.0–7.0)
Electrolyte imbalance	9 (10.5)	7.0 (3.0–11.5)
Anaemia	8 (9.3)	7.5 (2.5–13.0)
Shortness of breath	7 (80.1)	6.0 (3.0–20.0)
Dehydration	6 (7.0)	3.5 (1.75–8.5)
Chest pain	6 (7.0)	9.0 (2.5–19.5)
Urinary tract infection	5 (5.8)	3.0 (2.0–8.0)
Lethargy/Fatigue	4 (4.7)	3.0 (2.25–7.5)
General weakness	4 (4.7)	8.0 (2.25–18.25)
Pleural effusions	4 (4.7)	10.5 (4.0–14.75)
Decreased consciousness	3 (3.5)	14.0 (9–20)^c^
Confusion	3 (3.5)	8.0 (4–30)^c^
Bleeding	3 (3.5)	7.0 (2–8)^c^
Collapse	2 (2.3)	4 (4–4)^c^
Back pain	2 (2.3)	21.0 (14–28)^c^
Thrombocytopenia	2 (2.3)	17.0 (14–20)^c^
Tachycardia	2 (2.3)	4.0 (4–4)^c^
High creatinine	2 (2.3)	2.5 (2–3)^c^

^a^ Numbers add up to more than 86 as some patients were admitted with multiple symptoms and health conditions. Some of the symptoms may have developed shortly after the admission

^b^ Median length of stay when a patient admitted with a respective symptom or health condition. Patients may have been admitted with other concurrent symptoms or health conditions

^c^ Range reported

The final multivariable analysis identified the following variables as associated with unplanned presentations: cancer stage (stage 1 vs stage 4: IRR 2.50, 95% CI, 1.28–4.89; P = 0.01) and cancer type (lung cancer vs breast cancer: IRR 2.25, CI, 1.26–4.01; P = 0.01) (Table 4). There were seven multivariable models in total. The results from the univariable and multivariable analyses (Model 1 and the final model) are presented in Table 4. In Model 1, the variable intent of treatment had the highest overall p-value and was therefore removed in the subsequent model. Likewise, variables with the highest overall p-values were progressively removed in a backward stepwise manner, resulting in the final model. Among the 41 patients with lung cancer, 22 patients made 29 unplanned presentations (1 presentation by *n* = 15, and 2 presentations by *n* = 7). The most common reasons for these presentations were fever (*n* = 11), and shortness of breath (*n* = 8). Seventeen patients with lung cancer had 20 hospital admissions (1 admission by *n* = 14, and 2 admissions by *n* = 3). Common reasons included respiratory infection (*n* = 8), and fever (*n* = 11). Out of 13 patients with stage 1 cancer, 8 made 11 unplanned presentations (1 presentation by *n* = 5, and 2 presentations by *n* = 3), with fever (*n* = 5) as the most frequent reason.

**Table 4:** Multivariable predictors of unplanned presentation within the first three cycles of chemotherapy treatment

	Univariate analysis^b^			Multivariable analysis (Model 1)			Multivariable analysis (Final model)		
Predictor	IRR^a^	(95%CI)	P value	IRR^a^	(95%CI)	P value	IRR^a^	(95%CI)	P value
Gender									
Male	1.54	1.10–2.17	0.01	1.37	0.83–2.26	0.22			
Female	1.00			1.00					
Primary language									
English	0.71	0.48–1.06	0.09	0.79	0.53–1.19	0.26			
Other than English	1.00			1.00					
Working status			0.06			0.10			
Currently working	0.62	0.42–0.93	0.02	0.64	0.41–0.99	0.04			
Not in paid work	0.83	0.50–1.37	0.47	0.94	0.55–1.60	0.82			
Retired	1.00			1.00					
Cancer type			0.09			0.12			0.006
Colorectal cancer	0.87	0.44–1.72	0.69	0.74	0.33–1.66	0.47	0.87	0.43–1.73	0.69
Genitourinary cancer	1.05	0.54–2.03	0.88	0.67	0.28–1.60	0.37	1.05	0.52–2.10	0.90
Gynecological cancer	0.86	0.48–1.54	0.61	0.72	0.36–1.42	0.34	0.80	0.44–1.45	0.46
Lung cancer	2.23	1.34–3.72	0.002	1.39	0.67–2.90	0.38	2.25	1.26–4.01	0.01
Upper gastrointestinal cancer	1.63	0.98–2.72	0.06	1.23	0.61–2.46	0.57	1.65	0.94–2.90	0.08
Other	1.09	0.47–2.53	0.84	0.65	0.24–1.78	0.41	1.03	0.42–2.56	0.95
Breast cancer	1.00			1.00			1.00		
Cancer stage			0.02			0.001			0.01
Stage 1	1.92	1.02–3.64	0.05	3.50	1.47–8.29	0.01	2.50	1.28–4.89	0.01
Stage 2	0.61	0.35–1.06	0.08	0.73	0.34–1.58	0.43	0.71	0.39–1.29	0.26
Stage 3	0.81	0.52–1.26	0.35	1.08	0.57–2.04	0.82	0.99	0.62–1.59	0.97
Stage 4	1.00			1.00			1.00		
Chemotherapy history									
Received chemotherapy before	0.62	0.36–1.06	0.08	0.64	0.38–1.09	0.10			
First chemotherapy treatment	1.00			1.00					
Intent of treatment									
Palliative	1.32	0.92–1.89	0.13	1.36	0.69–2.68	0.37			
Curative	1.00			1.00					
Emetic risk			0.24			0.04			
Low	0.62	0.35–1.08	0.09	0.49	0.28–0.88	0.02			
Moderate	0.88	0.60–1.29	0.51	0.66	0.40–1.09	0.11			
High	1.00			1.00					

^a^ Incidence rate ratio, also adjusted for all variables shown and hospital site

^b^ Variables with any category having p < 0.20 were presented

## Discussion

The current study reported the incidence and risk factors for unplanned presentations, focusing on the initial treatment phase, specifically, the first three chemotherapy cycles. Nearly one-third of included patients experienced one or more unplanned presentations during the first three cycles of chemotherapy. Previous studies have reported the incidence of unplanned presentations during systemic treatment to range from 38 to 53% [6, 7, 19]. These data were typically collected within approximately one month following the administration of systemic therapy, without accounting for varying treatment timepoints (e.g., cycle 1, 2) or lengths (e.g., 21-day cycles). Therefore, direct comparison of these incidence rates with the findings of the current study is not possible due to differences in the timeframes used.

When using data on unplanned presentations during a fixed period (e.g., 30 days), the incidence of such clinical episodes may be influenced by the length of treatment the included patients receive. For example, if the sample includes a high number of cases with shorter treatment cycles (e.g., every 14 days), more chemotherapy would have been administered per patient within the specified period. This may explain why the incidence of unplanned presentations in the current study was lower than in previous studies [6, 7, 19].

The current study identified cancer stage and cancer type as risk factors for unplanned hospital presentations. Specifically, individuals with stage 1 cancer had a higher rate of unplanned presentations than those with stage 4 cancer. This may be explained by the high-dose chemotherapy typically administered in the early stages of cancer to maximize curative outcomes. In contrast, a study that identified cancer stage as a risk factor for emergency department visits during breast cancer treatment found that higher cancer stages were associated with an increased likelihood of such visits [19]. However, as this study did not include patients with stage 4 cancer, a direct comparison with the current findings is not appropriate. In our study, most Stage 4 patients who had treatment intent available (*n* = 124) had palliative intent treatment (*n* = 115), whereas most Stage 1, 2 and 3 had curative intent treatment. Additionally, as part of routine care, our participating hospitals referred patients receiving treatment with palliative intent to a community-based multidisciplinary palliative care team. A key component of this team's role was the management of symptoms and pain. This unique local context may help explain the relatively higher risk of unplanned hospital presentations among patients with Stage 1 cancer compared to those with Stage 4 disease.

Nausea and vomiting ranked as the second most common cause of unplanned presentations, highlighting the potential gap in side effect management support. Patients’ inadequate self-management of chemotherapy-induced nausea and vomiting is likely influenced by a range of contributing factors. These include patients’ perceptions that such side effects are expected and should therefore be endured [20], the cost of antiemetics [20], fears of treatment adjustments as a result of symptom reporting [21] and patients’ lack of understanding of the correct use of antiemetics [22]. There is potential for reducing unplanned presentations by enhancing the management of nausea and vomiting through the effective use of antiemetics and symptom monitoring. An example of a successful program is one that provided pharmacy-led support based on real-time remote monitoring of nausea and vomiting in patients receiving high-emetogenic risk regimens [23]. Their pre-post comparison demonstrated a decrease in unplanned healthcare utilization within 14 days of receiving chemotherapy.

Our study found that almost half of the unplanned presentations reported did not result in hospitalization, which is consistent with a previous study [24]. Direct discharge from the ED without hospital admission was considered a criterion for identifying preventable unplanned presentations in a study conducted by Shah and Neal [24], along with types of clinical care provided that could have been managed in an outpatient setting. Cases of direct discharges from the ED, as shown in our study, may indicate the potential for managing some of the side effects within the primary care or outpatient setting.

Consistent with a previous study [14], our study also found that common reasons for unplanned presentations included nausea and vomiting, diarrhea, and constipation, which were symptoms that could potentially be managed by primary care professionals such as general practitioners. Despite the potential role they could play in the early symptom management within the community, it is uncommon for patients to see GPs before making unplanned hospital admissions [25]. Opportunities exist in proactive monitoring of side effects using tools such as real-time symptom monitoring [26] and liaison with primary care for timely provision of low-acuity care closer to the patient’s home. The feasibility of training generalist community nurses to provide chemotherapy symptom management support through a shared care model has previously been demonstrated [27]. Further research is needed to examine ED clinical activity data to identify common types of care provided in the ED that could have been given in primary care.

Consistent with existing literature [14, 19, 28], our study showed that unplanned presentations were predominantly observed during cycle 1, which was slightly over half of all such clinical episodes observed within the first three cycles of chemotherapy. Previous studies [19, 28] have also identified receiving cycle 1 regime as an independent risk factor for making unplanned presentations. This is not surprising as clinicians typically implement strategies to mitigate risks in subsequent cycles, including dose adjustments, additional pharmacological support such as anti-emetics, and reinforcing patient education on symptom management. When designing targeted interventions to reduce unplanned presentations, such as active symptom monitoring, cycle 1 may therefore be the ideal time point for providing such support.

### Limitations and strengths of the study

This study used clinical trial data, which may report a lower incidence of unplanned presentations and hospitalization than cohort studies in routine practice [29]. The educational level of study participants was higher than the average Australian [30]. Additionally, although this study included patients who spoke English as a second language, they were proficient in written and spoken English to participate in the trial without needing an interpreter. Given that 42% of patients diagnosed with cancer within the local health district speak a language other than English at home, the generalizability of the findings may be limited [31]. Therefore, the study participants overall may have higher health literacy than the general population, limiting generalizability of the study findings. Furthermore, finding that patients with Stage 1 cancer had a higher risk of unplanned hospital presentations compared to those with Stage 4 cancer may reflect the unique local context. In this setting, patients receiving treatment with palliative intent were referred to a community-based multidisciplinary palliative care team. This context warrants caution when applying these findings to other clinical settings. The study was not powered to detect differences in the demographic and clinical predictors, and was limited to two metropolitan hospitals. Baseline characteristics were used as predictors to examine risk factors; therefore, some time-varying variables, such as treatment intent, cancer stage, and working status, were not reflected in our analysis. Finally, some variables, including treatment intent and cancer stage, were extracted from free-text clinical documentation, which contributed to missing data due to variability in recording practices. However, the current study reports the incidence of unplanned hospital presentations specifically at the beginning of chemotherapy treatment, providing valuable data for planning targeted interventions during this phase. It also accounts for time-at-risk in identifying risk factors, resulting in more accurate estimations, contributing to the existing evidence.

## Conclusion

This study reported the incidence and common reasons for unplanned presentations during the first three cycles of chemotherapy treatment and explored its associated risk factors. Strategies to improve the management of nausea and vomiting are warranted, given that they were the second most common reasons for unplanned presentations that could potentially be mitigated through improved symptom monitoring and antiemetic use. Such supportive care strategies may be most beneficial during the first cycle of chemotherapy treatment, when most unplanned presentations occurred. Finally, half of the unplanned presentations did not result in hospital admission, suggesting an opportunity to provide symptom management support through primary or outpatient services.

## Data Availability

The data used to support the findings of this study have not been made available due to ethics approval requirements.

## Declarations

### Trial registration

The study protocol of the original randomised controlled trial was registered at the Australian and New Zealand Clinical Trials Registry, for the trial ‘*Evaluating a shared-care pathway intervention to reduce chemotherapy outpatients' unplanned presentations to hospital – ESCAPI’*, which this secondary analysis was based on (registration number ACTRN12614001113640; registered on 21/10/2024: https://www.anzctr.org.au/Trial/Registration/TrialReview.aspx?id=367116&isReview=true).

## References

[1] Wilson BE, Jacob S, Yap ML, Ferlay J, Bray F, Barton MB (2019) Estimates of global chemotherapy demands and corresponding physician workforce requirements for 2018 and 2040: a population-based study. Lancet Oncol 20(6):769–780 31078462 10.1016/S1470-2045(19)30163-9

[2] Australian Institute of Health and Welfare. Cancer [Internet]. Canberra: Australian Institute of Health and Welfare; 2024 [cited 2025 Nov 14]. Available from: https://www.aihw.gov.au/reports/australias-health/cancer

[3] Pearce A, Haas M, Viney R, Pearson S-A, Haywood P, Brown C, Ward R (2017) Incidence and severity of self-reported chemotherapy side effects in routine care: a prospective cohort study. PLoS ONE 12(10):e0184360 29016607 10.1371/journal.pone.0184360 PMC5634543

[4] Kuderer NM, Desai A, Lustberg MB, Lyman GH (2022) Mitigating acute chemotherapy-associated adverse events in patients with cancer. Nat Rev Clin Oncol 19(11):681–697 36221000 10.1038/s41571-022-00685-3

[5] Lash RS, Hong AS, Bell JF, Reed SC, Pettit N (2022) Recognizing the emergency department’s role in oncologic care: a review of the literature on unplanned acute care. Emergency Cancer Care 1(1):6. 10.1186/s44201-022-00007-4 35844666 10.1186/s44201-022-00007-4 PMC9200439

[6] Tang M, Horsley P, Lewis CR (2018) Emergency department presentations in early stage breast cancer patients receiving adjuvant and neoadjuvant chemotherapy. Intern Med J 48(5):583–587. 10.1111/imj.13785 29722200 10.1111/imj.13785

[7] Dufton PH, Drosdowsky A, Gerdtz MF, Krishnasamy M (2019) Socio-demographic and disease related characteristics associated with unplanned emergency department visits by cancer patients: a retrospective cohort study. BMC Health Serv Res 19(1):647. 10.1186/s12913-019-4509-z 31492185 10.1186/s12913-019-4509-z PMC6731557

[8] Australian Institute of Health and Welfare. (2022). Cancer. Australian Government. Retrieved from https://www.aihw.gov.au/reports/australias-health/cancer. Accessed March 2026

[9] Fethney J, Kim B, Boustany C, McKenzie H, Hayes L, Cox K, Simpson JM, Horvath LG, Vardy JL, McLeod J (2024) Evaluating a shared care pathway intervention for people receiving chemotherapy to reduce post-treatment unplanned hospital presentations: a randomised controlled trial. Support Care Cancer 32(1):77 38170289 10.1007/s00520-023-08261-w PMC10764538

[10] Lee H-O, Lee J-J (2015) Nutritional intervention using nutrition care process in a malnourished patient with chemotherapy side effects. Clin Nutr Res 4(1):63–67 25713794 10.7762/cnr.2015.4.1.63 PMC4337925

[11] Jordan K, Gralla R, Jahn F, Molassiotis A (2014) International antiemetic guidelines on chemotherapy induced nausea and vomiting (CINV): content and implementation in daily routine practice. Eur J Pharmacol 722:197–202 24157984 10.1016/j.ejphar.2013.09.073

[12] Andreyev J, Ross P, Donnellan C, Lennan E, Leonard P, Waters C, Wedlake L, Bridgewater J, Glynne-Jones R, Allum W (2014) Guidance on the management of diarrhoea during cancer chemotherapy. Lancet Oncol 15(10):e447–e460 25186048 10.1016/S1470-2045(14)70006-3

[13] Yang W, Williams JH, Hogan PF, Bruinooge SS, Rodriguez GI, Kosty MP, Bajorin DF, Hanley A, Muchow A, McMillan N (2014) Projected supply of and demand for oncologists and radiation oncologists through 2025: an aging, better-insured population will result in shortage. J Oncol Pract 10(1):39–45 24443733 10.1200/JOP.2013.001319

[14] Bright HR, Chandy SJ, Chacko RT, Backianathan S (2019) Intercycle unplanned hospital admissions due to cisplatin-based chemotherapy regimen-induced adverse reactions: a retrospective analysis. Curr Drug Saf 14(3):182–191. 10.2174/1574886314666190619123047 31250766 10.2174/1574886314666190619123047 PMC6865053

[15] Fessele KL, Hayat MJ, Atkins RL (2017) Predictors of unplanned hospitalizations in patients with nonmetastatic lung cancer during chemotherapy. Oncol Nurs Forum 44(5):E203-e212. 10.1188/17.Onf.E203-e212 28820513 10.1188/17.ONF.E203-E212 PMC5856246

[16] Dufton PH, Gerdtz MF, Jarden R, Krishnasamy M (2022) Methodological approaches to measuring the incidence of unplanned emergency department presentations by cancer patients receiving systemic anti-cancer therapy: a systematic review. BMC Med Res Methodol 22(1):75. 10.1186/s12874-022-01555-3 35313807 10.1186/s12874-022-01555-3 PMC8935762

[17] von Elm E, Altman DG, Egger M, Pocock SJ, Gøtzsche PC, Vandenbroucke JP (2007) The strengthening the reporting of observational studies in epidemiology (STROBE) statement: guidelines for reporting observational studies. Ann Intern Med 147(8):573 17938396 10.7326/0003-4819-147-8-200710160-00010

[18] Berger MJ, Ettinger DS, Aston J, Barbour S, Bergsbaken J, Bierman PJ, Brandt D, Dolan DE, Ellis G, Kim EJ, Kirkegaard S, Kloth DD, Lagman R, Lim D, Loprinzi C, Ma CX, Maurer V, Michaud LB, Nabell LM, Noonan K, Roeland E, Rugo HS, Schwartzberg LS, Scullion B, Timoney J, Todaro B, Urba SG, Shead DA, Hughes M (2017) NCCN guidelines insights: antiemesis, version 2.2017. J Natl Compr Canc Netw 15(7):883–893. 10.6004/jnccn.2017.0117 28687576 10.6004/jnccn.2017.0117

[19] Pittman NM, Hopman WM, Mates M (2015) Emergency room visits and hospital admission rates after curative chemotherapy for breast cancer. J Oncol Pract 11(2):120–125. 10.1200/jop.2014.000257 25585617 10.1200/JOP.2014.000257

[20] Childs DS, Looker S, Le-Rademacher J, Ridgeway JL, Breitkopf CR, Jatoi A (2019) What occurs in the other 20% of cancer patients with chemotherapy-induced nausea and vomiting (CINV)? A single-institution qualitative study. Support Care Cancer 27(1):249–255. 10.1007/s00520-018-4323-x 29938306 10.1007/s00520-018-4323-x PMC6309746

[21] Farrell C, Heaven C (2018) Understanding the impact of chemotherapy on dignity for older people and their partners. Eur J Oncol Nurs 36:82–88 30322514 10.1016/j.ejon.2018.05.008

[22] Aapro M, Ruffo P, Panteri R, Costa S, Piovesana V (2018) Oncologist perspectives on chemotherapy-induced nausea and vomiting (CINV) management and outcomes: a quantitative market research-based survey. Cancer Rep 1(4):e1127. 10.1002/cnr2.1127 10.1002/cnr2.1127 PMC7941545 32729252

[23] Hough S, McDevitt R, Nachar VR, Kraft S, Brown A, Christen C, Frame D, Smerage JB (2021) Chemotherapy remote care monitoring program: integration of SMS text patient-reported outcomes in the electronic health record and pharmacist intervention for chemotherapy-induced nausea and vomiting. JCO Oncol Pract 17(9):e1303–e1310 33534634 10.1200/OP.20.00639

[24] Shah MP, Neal JW (2021) Relative impact of anticancer therapy on unplanned hospital care in patients with non–small-cell lung cancer. JCO Oncol Pract 17(8):e1131–e1138. 10.1200/op.20.00612 33351677 10.1200/OP.20.00612

[25] Cuppens K, Oyen C, Derweduwen A, Ottevaere A, Sermeus W, Vansteenkiste J (2016) Characteristics and outcome of unplanned hospital admissions in patients with lung cancer: a longitudinal tertiary center study. Towards a strategy to reduce the burden. Support Care Cancer 24(7):2827–2835. 10.1007/s00520-016-3087-4 26816091 10.1007/s00520-016-3087-4

[26] Basch E, Deal AM, Kris MG, Scher HI, Hudis CA, Sabbatini P, Rogak L, Bennett AV, Dueck AC, Atkinson TM (2016) Symptom monitoring with patient-reported outcomes during routine cancer treatment: a randomized controlled trial. J Clin Oncol 34(6):557 26644527 10.1200/JCO.2015.63.0830 PMC4872028

[27] Kim B, Boustany C, Acret L, McLeod J, Cook N, McKenzie H, Hayes L, Fethney J, Simpson JM, Willcock S (2024) Perspectives and experiences of healthcare professionals involved in a community nurse‐delivered shared care model intervention designed to support outpatients receiving chemotherapy: a qualitative study using interviews. Eur J Cancer Care 2024(1):2206346

[28] Loerzel VW, Hines RB, Deatrick CW, Geddie PI, Clochesy JM (2021) Unplanned emergency department visits and hospital admissions of older adults under treatment for cancer in the ambulatory/community setting. Support Care Cancer 29(12):7525–7533. 10.1007/s00520-021-06338-y 34105026 10.1007/s00520-021-06338-y

[29] Prince RM, Powis M, Zer A, Atenafu EG, Krzyzanowska MK (2019) Hospitalisations and emergency department visits in cancer patients receiving systemic therapy: systematic review and meta-analysis. Eur J Cancer Care 28(1):e12909. 10.1111/ecc.12909 10.1111/ecc.12909 30238542

[30] Australian Bureau of Statistics. Education and training: Census, 2021 [Internet]. Canberra: Australian Bureau of Statistics; 2022 Dec 09 [cited 2025 Nov 14]. Available from: https://www.abs.gov.au/statistics/people/education/education-and-training-census/2021

[31] Cancer Institute NSW. A snapshot of linguistically diverse people diagnosed with cancer [Internet]. Sydney: Cancer Institute NSW; 2024 [cited 2025 Nov 14]. Available from: https://www.cancer.nsw.gov.au/getattachment/c85ca036-a889-4096-9f1f-28814f6719eb/snapshot-of-cald-people-with-cancer.pdf

[32] Kim B, Boustany C, Acret L, McLeod J, Cook N, McKenzie H, Hayes L, Fethney J, Simpson JM, Willcock S (2024) Perspectives and experiences of healthcare professionals involved in a community nurse-delivered shared care model intervention designed to support outpatients receiving chemotherapy: a qualitative study using interviews. Eur J Cancer Care. 10.1155/2024/2206346

[33] Hanna TP, King WD, Thibodeau S, Jalink M, Paulin GA, Harvey-Jones E, O’Sullivan DE, Booth CM, Sullivan R, Aggarwal A (2020) Mortality due to cancer treatment delay: systematic review and meta-analysis. BMJ 371 10.1136/bmj.m4087 PMC7610021 33148535

[34] Liu H, Liu B, Zheng F, Chen X, Ye L, He Y (2020) Distribution of pathogenic bacteria in lower respiratory tract infection in lung cancer patients after chemotherapy and analysis of integron resistance genes in respiratory tract isolates of uninfected patients. J Thorac Dis 12(8):4216 32944333 10.21037/jtd-20-928 PMC7475539

[35] Hsu-Kim C, Hoag JB, Cheng G-S, Lund ME (2013) The microbiology of postobstructive pneumonia in lung cancer patients. J Bronchol Interv Pulmonol. 10.1097/LBR.0b013e31829ddf01 10.1097/LBR.0b013e31829ddf01 23857204

[36] Qiao D, Wang Z, Lu Y, Wen X, Li H, Zhao H (2015) A retrospective study of risk and prognostic factors in relation to lower respiratory tract infection in elderly lung cancer patients. Am J Cancer Res 5(1):423–432 25628950 PMC4300720

[37] Taplitz RA, Kennedy EB, Bow EJ, Crews J, Gleason C, Hawley DK, Langston AA, Nastoupil LJ, Rajotte M, Rolston KV, Strasfeld L, Flowers CR (2018) Antimicrobial prophylaxis for adult patients with cancer-related immunosuppression: ASCO and IDSA clinical practice guideline update. J Clin Oncol 36(30):3043–3054. 10.1200/jco.18.00374 30179565 10.1200/JCO.18.00374

[38] Jairam V, Lee V, Park HS, Thomas CR, Melnick ER, Gross CP, Presley CJ, Adelson KB, James BY (2019) Treatment-related complications of systemic therapy and radiotherapy. JAMA Oncol 5(7):1028–1035 30946433 10.1001/jamaoncol.2019.0086 PMC6583836

[39] Kogan LG, Davis SL, Brooks GA (2019) Treatment delays during FOLFOX chemotherapy in patients with colorectal cancer: a multicenter retrospective analysis. J Gastrointest Oncol 10(5):841–846. 10.21037/jgo.2019.07.03 31602321 10.21037/jgo.2019.07.03 PMC6776798

[40] Adriani M, Syahrir M, Andayani YD, Djamaluddin N (2023) Update management of chemotherapy-induced neutropenia: a narrative literature review. Bioscientia Medicina: Journal of Biomedicine and Translational Research 7(1):3068–3073

[41] Aapro MS, Chaplin S, Cornes P, Howe S, Link H, Koptelova N, Mehl A, Di Palma M, Schroader BK, Terkola R (2023) Cost-effectiveness of granulocyte colony-stimulating factors (G-CSFs) for the prevention of febrile neutropenia (FN) in patients with cancer. Support Care Cancer 31(10):581. 10.1007/s00520-023-08043-4 37728795 10.1007/s00520-023-08043-4 PMC10511548

